# *Amorphophallus konjac*: Sensory Profile of This Novel Alternative Flour on Gluten-Free Bread

**DOI:** 10.3390/foods11101379

**Published:** 2022-05-10

**Authors:** Fernanda Laignier, Rita de Cássia de Almeida Akutsu, Bernardo Romão de Lima, Renata Puppin Zandonadi, António Raposo, Ariana Saraiva, Raquel Braz Assunção Botelho

**Affiliations:** 1 Human Nutrition Post-Graduate Program, College of Health Sciences, University of Brasília, Brasília 70910900, DF, Brazil; felaignier@hotmail.com (F.L.); rita.akutsu@gmail.com (R.d.C.d.A.A.); bernardo.lima@aluno.unb.br (B.R.d.L.); renatapz@unb.br (R.P.Z.); 2 Department of Nutrition, College of Health Sciences, University of Brasília, Brasília 70910900, DF, Brazil; 3 CBIOS (Research Center for Biosciences and Health Technologies), Universidade Lusófona de Humanidades e Tecnologias, Campo Grande 376, 1749-024 Lisboa, Portugal; 4 Department of Animal Pathology and Production, Bromatology and Food Technology, Faculty of Veterinary, Universidad de Las Palmas de Gran Canaria, Trasmontaña s/n, 35413 Arucas, Spain; ariana_23@outlook.pt

**Keywords:** check-all-that-apply (CATA), glucomannan, hedonic scale, bread, gluten-free

## Abstract

This study aimed to evaluate the sensory profile of gluten-free bread with *Amorphophallus konjac* (AK) flour in different concentrations. This experimental study is divided into three steps: preparation of the gluten-free bread formulations, sensory analysis, and statistical analysis. The addition of Konjac flour in a gluten-free bread formulation was tested in different proportions, 12.5%, 25%, 37.5%, and 50% of the flour content. The checking all-that-apply (CATA) was conducted with 110 panelists; among these, 43 were consumers of gluten-free bread. Sensory analysis was conducted using a 9-point hedonic scale for color, aroma, texture, flavor, appearance, and overall acceptability. The AK flour influenced the sensory characteristics of gluten-free bread. Bread with characteristics closer to those found in bread with gluten was the one with 12.5% of konjac flour for both the acceptability analysis as the attributes raised through a detailed CATA map. The control sample is located next to features like dry appearance, dry texture and grainy, dark color, and salty. Therefore, 12.5% AK gluten-free bread is closer to the characteristics of the control sample, such as light crust color, light crumb color, soft and moist texture, cohesion, and brightness. The bread with the highest percentage of overall consumer acceptance was 12.5% konjac with 93% and 96% acceptance among consumers and non-consumers of gluten-free bread, respectively.

## 1. Introduction

Bread is a worldwide staple food, present in many civilizations since most antique eras, and is still largely consumed today. Its consumption revolves around 24 kg per capita/year worldwide, with evident growth trends for consumption and market value [1]. A similar tendency is noted regarding the dietary habits of adepts of a strict gluten-free diet (GFD), with bread highlighted as one of the most demanded and consumed gluten-free products [2,3]. However, gluten withdrawal is a challenge in bread formulations since texture, color, crumb, and alveoli structure are sensory and technological characteristics that rely mainly on gluten’s presence and strength [4]. Thus, with gluten removal, impairments related to these characteristics are noteworthy and impact overall sensory aspects [5].

Gluten-free bread tends to present reduced volume and alveoli structure, brittle texture, light crust color, and usually, loaves present heavy, tenacious, and elastic mouthfeel [6,7]. Ingredients such as hydrocolloids, protein, and dietary fiber have been used to mimic gluten viscoelastic characteristics. However, although acceptance might be improved, the overall characteristics are still very apart from its gluten-containing counterpart [7,8].

Among the studied alternatives, *Amorphophallus konjac* (AK) is a mountain-based plant native to southern Asia and Africa, with numerous corms from which a water-soluble, non-cellulosic polysaccharide of high molecular weight is extracted [9]. Glucomannan is the AK main polysaccharide, usually applied to pharmaceuticals, cosmetics, and some food products, such as wheat noodles and bread [10,11]. AK’s glucomannan flour was successfully tested as a starch- and gluten-replacer in gluten-free noodles due to its rheological and viscoelastic characteristics [12]. Because of its success in replacing wheat in noodles, some studies evaluated the AK’s glucomannan as an additive to gluten-free bread [13,14,15].

Laignier et al. [16] tested AK flour’s use in high amounts (up to 50% of flour) on gluten-free bread’s nutritional and physicochemical properties. Despite the successful use of AK flour in gluten-free bread’s nutritional and physicochemical aspects [16], sensory analysis was not performed on this product, and it is crucial to encourage consumption. Therefore, this study aimed to evaluate the sensory profile of gluten-free bread with *Amorphophallus konjac* flour in different concentrations.

## 2. Materials and Methods

This exploratory and quantitative study was performed in two steps: (I) Sample preparation, and (II) Sensory Analysis. The Check-all-that-apply test (CATA) was used since it is a rapid descriptive method that serves as a simple and effective alternative to screen the sensory attributes and drivers of liking various products [17]. In addition, we used the hedonic scale since it can measure the product’s sensory perception and the intensity of its attributes [18]. Therefore, combining these two methods might result in a complete screening of the sensory profile of foods.

### 2.1. Sample Preparation

The samples were developed at the University of Brasilia, Federal District, Brazil, according to the formulations developed by Laignier et al. [16]. The control gluten-free bread (GFB) was composed of gluten-free flour (30% of potato starch and 70% of rice flour), sucrose (12 g/100 g of control flour basis), salt (3 g/100 g of control flour basis); water (34.5 g/100 g of control flour basis), soy oil (16.5 g/100 g of control flour basis), whole egg (29.5 g/100 g of control flour basis), and yeast (1.5 g/100 g of control flour basis). The same ingredients were used in the modified bread samples added of konjac flour in the proportion of 12.5%, 25%, 37.5%, and 50% of the gluten-free flour amount [16]. According to the supplier (SM pharmaceutical enterprises, imported by Almofariz Pharmaceuticals), AK flour was composed of 70% glucomannan konjac and obtained in three different lots (18F13-B022-034221, 18F13-B022-034226, MW20171011-1750).

The water content was adjusted due to the AK flour’s capacity to absorb more water than the gluten-free flour used in control bread. Therefore, the water was used in 131%, 228%, 297%, and 406%, based on the weight of the flour basis. The formulations can be accessed in Table S1—Supplementary File. The ingredients were weighed on a 0.2 g precision scale (Ohaus^®^, Parsippany, NJ, USA), and the yeast was pre-activated with sucrose and warm water (38 °C) for 10 min. Rice flour, potato starch, konjac flour (in modified samples), and salt were mixed in another recipient. Afterwards, eggs, water, and oil were mixed with the dry ingredients. The activated yeast was added, and the dough was kneaded and rested for 50 min (27 °C). Afterwards, the dough went through a second kneading and modeling (40 g spheres). Finally, the samples were baked for 40 min. in a preheated (180 °C) gas oven Brastemp^®^-Sau Paulo, Brazil [16].

**Figure 1:**
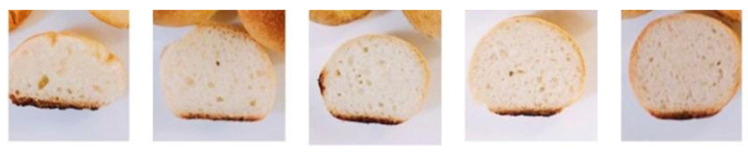
Gluten-free bread—from left to right, control gluten-free bread; bread with the addition of 12.5% of *Amorphophallus konjac* flour; 25%; 37.5%; and 50%.

After baking, the samples were removed from the trays and cooled down in polyethylene containers. Bread samples were codified with 3-digit random numbers (Figure 1). Samples were served the same day they were baked, portioned in slices of 10 g each. They were served in disposable white plastic plates and two water glasses, one full with room temperature water and another empty for occasional disposal of samples.

### 2.2. Sensory Analysis

First, the sensory descriptors for CATA were elaborated by applying the Kelly’s Repertory Grid Method [19] with five expert panel members, all with 240 h minimum experience related to gluten-free products’ development. None of the above experts were part of the following sensory test. Three sessions lasting 30-min each were held, and with the help of an expert panel moderator, 30 descriptors were raised by the grid method (7 for appearance—dark crust color, dry crust appearance, light crust color, light crumb color, dark crumb color, pretty, and shiny; 9 for flavor—seafood flavor, yeast flavor, unpleasant flavor, pleasant flavor, bitter, sour, sweet flavor, astringent, savory flavor; 9 for texture—moist, stiff, sticky, cohesive, rubbery, soft, compact, buttery, grainy, dry; and 5 for aroma—egg scent, fried food scent, seafood scent, yeast scent, and pleasant scent).

Bread samples were also submitted to an evaluation using a 9-point hedonic scale (1 = dislike extremely; 2 = dislike very much; 3 = dislike moderately; 4 = dislike slightly; 5 = neither like nor dislike; 6 = like slightly; 7 = like moderately; 8 = like very much; 9 = like extremely) to measure the acceptance for appearance, flavor, texture, aroma, and global acceptance [20,21]. Both CATA and acceptance tests were performed on the same day, simultaneously, starting at 9 am, in the sensory evaluation laboratory at the University of Brasilia, Brazil. The study was approved by a Brazilian Ethics Committee (CAAE: 01154818.7.0000.0030), and before the tests, participants signed a consent form. The recruitment questionnaire collected demographic information, and regular consumption of any bread and willingness to collaborate were prerequisites to participate. In the questionnaire, we also asked about the regular consumption of gluten-free bread.

Both CATA descriptors and samples’ order were presented in a randomized and balanced order in the sensory forms. Samples were presented monadically. The panelists answered the forms according to their perception of the suggested descriptors’ presence and the attributes’ intensity.

110 untrained consumers participated for both simultaneous tests, and 43 (39%) were regular gluten-free bread consumers. Of the 110 participants, 68.18% were female, and 31.82% were male, from 18 and 59 years. Participants were recruited through invitations posted on social media. The individuals who arrived at the laboratory through the invitation to perform the sensory test and met the inclusion criteria were included in the sample without gender balance.

### 2.3. Statistic Analysis

Two groups were formed to overview the differences between the perception of all consumers—one with all the consumers (Group I; *n* = 110) and another with only regular consumers of gluten-free bread (Group II; *n* = 43). The acceptance percentage was obtained from the scores given by the panelists in the acceptance test. A product was considered accepted when it obtained at least 70% approval (6–9 on a hedonic scale) [22].

A two-way ANOVA followed by Tukey’s test (95%; *p* < 0.05) was performed to compare the attributes evaluated in the hedonic scale acceptance test. SPSS (IBM^®^ Corp, Armonk. NY, USA, 2015) was used in this step.

For the CATA analysis, descriptors were initially compared with non-parametric Cochran’s Q test (*p* < 0.0001) to assess whether there were significant differences in consumers’ perception of any given attribute among the different samples. Then, a multiple pairwise comparison test was performed using Bonferroni (McNemar) procedure with 5% significance. A test of independence between rows and columns was carried out with 5% of significance. Additionally, correspondence analysis (CA) based on chi-squared distances was performed to summarize a sensory map of samples.

## 3. Results

### 3.1. Hedonic Scale Acceptance Test

Table 1 presents the acceptance of gluten-free bread samples evaluated by all participants (*n* = 110) with a hedonic scale. Gluten-free bread that used 12.5% AK flour replacing part of the gluten-free flour presented the highest mean for appearance, texture, and global acceptance. Regarding flavor and aroma, this sample (12.5%) and the control also presented the highest means.

Table 2 shows the acceptance percentage of the different formulations of gluten-free bread among the 43 consumers of gluten-free bread and the 67 non-consumers of gluten-free bread. Among consumers of gluten-free bread, the 12.5 of AK flour sample presented the highest percentual of acceptance considering appearance, texture, and global acceptance. Among non-consumers of gluten-free bread, the 12.5 of AK flour sample presented the highest percentual of acceptance for all attributes. Flavor and aroma acceptance was higher for the control sample in the group of gluten-free bread consumers.

### 3.2. Check-All-That-Apply (CATA)

A total of 30 CATA descriptors were raised after the Repertory Grid method. About 66% (*n* = 20) were perceived as different among all samples by the consumers of Group I (all consumers; *n* = 100) (*p* < 0.0001; Table 3). Almost half (46.6%; *n* = 14) of the descriptors were perceived as different by members of Group II (gluten-free bread consumers; *n* = 43) (*p* < 0.0001; Table 4).

In general, for both groups, the most used descriptors to evaluate AK gluten-free bread developed by this study were: “Light Crust Color”, “Light Crumb Color”, “Pretty”, “Pleasant Scent”, “Rubbery”, “Plesant Flavor”, “Yeast Flavor”, “Unpleasant Flavor”, “Sweet Flavor”, and “Soft”, however, a very heterogeneous distribution among the frequency of descriptors between all samples was evident. Regarding the appearance of AK Gluten-free bread, for both groups, descriptors such as “Light Crust Color”, “Light Crumb Color”, and “Pretty” were the most frequent ones. In group I, differences in sample’s appearance were found in six descriptors: “Dark Crust Color”, “Dry Crust Appearence”, “Light Crust Color”, “Light Crumb Color”, “Dark Crumb Color”, and “Pretty” (Table 3). In Group II, differences were found for the same descriptors, except for “Dark Crust Color” and “Light Crumb Color” (Table 4).

The term “Dark crust color” was most frequent in sample 12.5; however, no differences were found compared to sample 50, while differences were present in the remaining ones. Sample 12.5 (Group I) presented the highest frequency of the term “Dry Crust Appearance”, while in Group II, the same was also true, while all the remaining samples did not present differences between them.

“Light crust color” was more frequent in sample 25, in group I, and equal in frequency in samples 25 and 37.5 in group II; however, differences between all samples were present in both groups. The term “Dark Crumb Color” was most frequent in sample 50; however, in both groups, sample 25 was not significantly different for the same term, although presenting a lower absolute frequency proportionally. The control sample presented a higher frequency for the term “Pretty” in all groups, though, in group I, sample 12.5 did not show significant differences for the same term.

Regarding the aroma of bread, in both groups, “Pleasant scent” presented the highest frequency among all samples, given that sample 12.5 presented the highest value for this descriptor. However, no differences were seen between samples 12.5 and control in group I, while in group II, sample 12.5 was different from all the remaining others. As for the descriptor “Seafood scent,” sample 12.5 was the only significantly different from the others in both groups.

“Pleasant flavor” and “Unpleasant flavor” presented similar distributions in both groups. Samples 12.5 and control did not statistically differ, while samples 25, 37.5, and 50 were in the same group, with a “Seafood flavor” also highlighted as a frequent descriptor. Samples 12.5 and control presented no differences while the other samples were in the same group. In group I, “Yeast flavor” was a frequent descriptor, with significant differences between samples. In group II, no differences for the same descriptor were found.

The control sample presented the highest frequency for “Sweet flavor” in groups I and II. In group I, no difference was found between control and 12.5%. In group II, differences were present only between control and 25%. The highest frequencies for “Savory flavor” were in the control sample in both groups. Differences were found only between control and 50%.

Regarding texture-related descriptors, control presented the highest frequencies for “Dry”, “Grainy”, “Stiff”, “Compact”, and “Buttery” in both groups I and II. The term “Moist” was most frequent in samples 12.5% and 37.5% in group I and 37.5% and 50% in group II. The control sample presented the lowest frequency for this descriptor in both groups.

In group I, “Stiff” was most frequent in the control sample, presenting differences compared to all remaining samples. In group II, sample 25% was statistically equal, although showing a much lower frequency. “Rubbery” was present in samples 25%, 37.5%, and 50%, with no significant differences between these samples in both groups.

“Soft” was frequent in sample 12.5%, presenting differences in all remaining samples in groups I and II. The remaining samples did not present differences between them for the same descriptor. In groups I and II, the descriptor “Rubbery” did not present differences regarding control and 12.5%, while samples 25%, 37.5%, and 50% were different from these other two and statistically the same.

A heterogeneous distribution was evidenced for “Grainy”. The control sample presented the highest frequency for this term in both groups. However, in group I, control and 50% did not present differences, while in group II, control differed only from the sample 12.5%. The descriptor “Dry” was most frequent in control, differing from all remaining samples in all groups.

**Figure 2:**
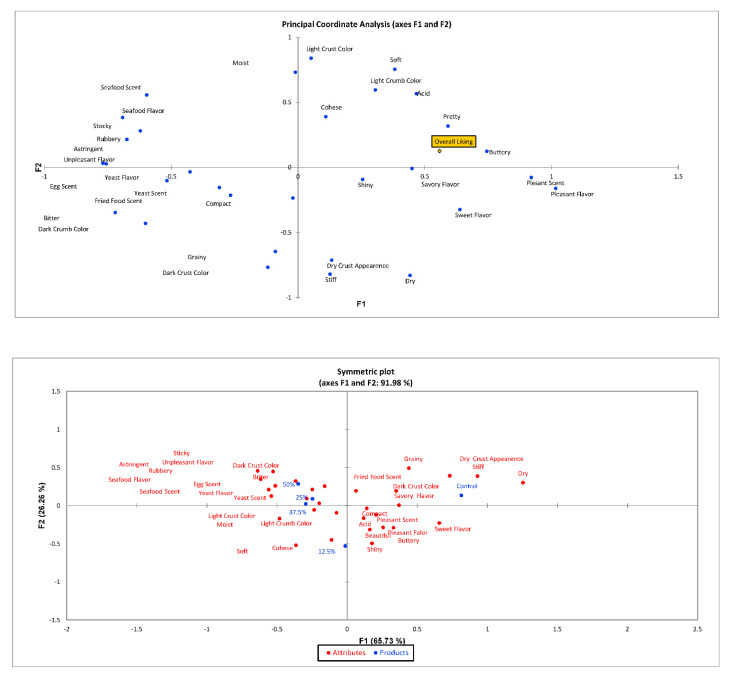
Sensory descriptive map resulting from Correspondence Analysis performed on the CATA data (*n* = 110).

**Figure 3:**
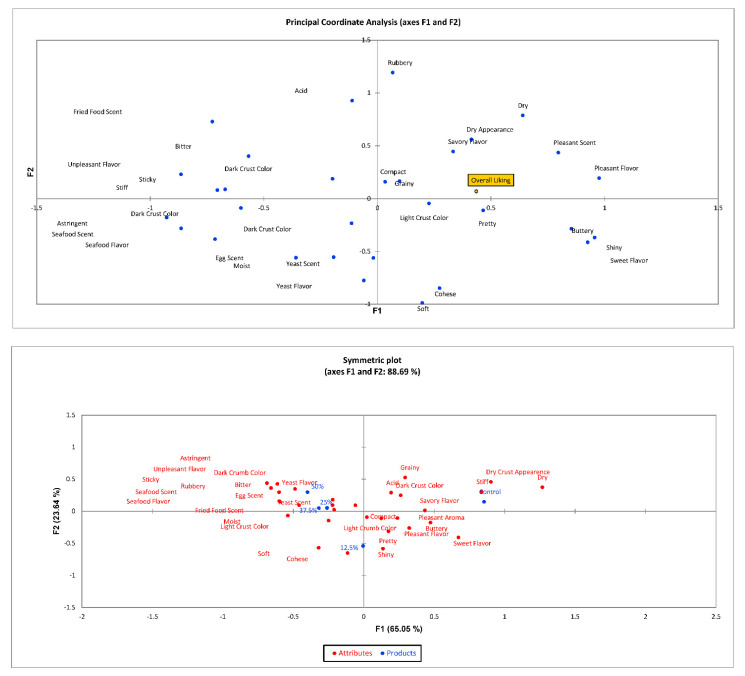
Sensory descriptive map resulting from Correspondence Analysis performed on the CATA data (*n* = 43).

Correspondence analysis was carried out to generate a sensory map of samples and their relation with attributes. Groups 1 and 2 Principal Coordinate Analysis (PCA) and Symmetric Plot are in Figure 2 and Figure 3.

## 4. Discussion

Hydrocolloids like AK flour in gluten-free bread are used to mimic viscoelastic and crumb texture performed by gluten to improve sensory characteristics. Our study first evaluated the sensory profile of gluten-free bread using *Amorphophallus konjac* flour in higher proportions (12.5–50%) of gluten-free flour, which makes it difficult to compare the results with other studies. *Amorphophallus konjac* flour in proportions of 12.5%, 25%, 37.5%, and 50% of the gluten-free flour content was previously tested in gluten-free bread, but only the nutritional and physicochemical properties were evaluated [16].

A study evaluated the acceptance of gluten-free bread with AK glucomannan but used in lower amounts [13]. The authors used rice and potato flour and corn and cassava starch as gluten-free flour matrices. The AK flour was used combined, with xanthan gum as an additive [13]. The product was evaluated using the 9-point hedonic scale. The increasing proportion of AK flour and the decreasing proportion of xanthan gum in the formulation decreased the panelists’ preference. The highest-rated gluten-free bread was that with xanthan gum and AK flour proportion of 0.25: 0.75 [13]. Considering the 9-point hedonic scale, the lowest addition of AK flour (12.5%) presented the best acceptance. Considering appearance, 12.5% gluten-free bread had the highest average, different from other formulations. This formulation also had the best scores for flavor, texture, and global acceptance, similar to the control bread only for the flavor and aroma attributes.

Regarding flavor, bread with percentages above 25% presented lower acceptance means than control and 12.5%. Therefore, the addition of konjac flour negatively influenced the flavor of bread above 12.5%. This could be explained by the water absorption capacity of AK flour. The final volume of the dough increases because of the need for water addition. It decreases the concentration of ingredients that provide greater palatability to food, such as sugar, fat, and salt [23]. A potential influence of seafood flavor showed by CATA test could also explain this result. The more AK flour was added to formulations, the more seafood flavor was observed on samples (Table 3 and Table 4). AK presents a seafood-like flavor mainly due to trimethylamine (TMA), a nitrogenous base aliphatic organic compound. Although AK flour has many advantages in food application, the fish-like flavor is the primary factor limiting its application in some food. The food industry has been searching for alternatives to reduce it [24]. It was also confirmed by CATA test, in which the bread with the highest score for the term pleasant flavor was 12.5%, and for unpleasant flavor, 50%, indicating the possible influence of AK flour on the gluten-free bread flavor. The result of CATA agrees with the result obtained in the sensory analysis, in which bread 12.5% had the highest average for flavor and bread 50% had the lowest average.

There was no difference in aroma acceptance between the control bread and the 12.5% in this study. However, there is a difference between the control bread and the bread with AK flour percentages above 37.5%, indicating that the higher the percentage of AK flour less accepted the aroma of the gluten-free bread. This was probably due to the characteristic odor of the AK flour or the lower concentration of sugars and proteins, which prevented chemical reactions that produce aroma, such as the Maillard reaction and caramelization, from adequately occurring. Regarding the aroma of konjac bread, some comments could be extracted from the evaluation forms filled in by the evaluators, “Despite the smell of fish, it has a normal bread texture”; “although there is a smell of seafood, the taste is good”; “Taste good, but bad aroma”. This was confirmed by the CATA test, in which “Seafood Scent” was most frequent among samples with 25% to 50% of AK flour addition. Considering CATA, the control bread had the lowest score for seafood aroma, and in the AK flour bread, more AK flour led to more consumers perceiving this odor. The 50% bread was most punctuated for seafood aroma, and, in the sensory analysis, 50% AK flour bread had the lowest average for aroma. This can be explained by the characteristic odor of konjac flour that resembles a fish [25]. In the CATA test for aroma, attributes such as egg, frying, and yeast aroma were mentioned and considered statistically equal for all samples. The pleasant aroma was statistically equal and better evaluated in control and 12.5% samples.

The bread samples were considered statistically equal in texture, except for the 12.5% bread which presented the highest acceptance rate. This bread sample was the one that presented the highest instrumental hardness in a previous study [16]; however, in the acceptance test, it was the bread that obtained the highest average in the consumers’ evaluation. Despite Bourne’s [26] assertion that texture is the main attribute considered to reject food, we observed that bread with greater firmness [16] was also well accepted by consumers. These results corroborate the findings of another study [27] that obtained bread with high means of instrumental hardness but were well accepted by trained evaluators. In a previous study [16], the 50% AK flour bread had one of the lowest means of instrumental hardness (e.g., the softest bread). However, it presents the lowest percentage of texture acceptance among all consumers and gluten-free bread consumers. Analyzing the term sticky, the 50% AK bread obtained the highest frequency, differing from the control bread and 12.5%, with the control being the least sticky. This may also have influenced the best acceptance of this product compared to the others. Additionally, the bread samples considered rubberier were those with 25% or more AK flour, and the less rubbery ones, without statistically differing, were the control bread and the 12.5% bread.

The sample with 12.5% of AK flour obtained a percentage of global acceptance among evaluators who habitually consume gluten-free bread of 93.03% and among non-consumers of 95.52%. This result was the opposite of that obtained by another study [25]. Gluten-free bread was more accepted among evaluators from the celiac group who habitually consumed these foods than among non-consumers of gluten-free bread. Our result is important, showing that gluten-free bread prepared with 12.5% AK flour is well accepted by all consumers, and it could be essential to insert this product on the market.

Considering the CATA test, the data show that the dark color crust obtained a significantly higher frequency for the control bread, reflecting the same result obtained in the instrumental analysis in a previous study, indicating that this was the darkest crust color bread [16]. The possible presence of components can explain this in adequate amounts (higher concentrations than the other samples) for the occurrence of chemical reactions of non-enzymatic browning in foods. The visual presentation of a product marks the first contact with the consumer; therefore, characteristics such as color and appearance are frequently observed and are associated with personal reactions of acceptance, indifference, or rejection [28], as observed in the 9-point hedonic scale acceptance.

The term dry appearance was considerably more frequent for the control bread, which was statistically different from the others. The least pointed to this term was 12.5% bread, which was not expected since our previous study showed that, among AK flour samples, the 12.5% presented the lowest moisture content [16]. However, it was evident that the addition of AK flour in 12.5% of gluten-free flour improved the appearance of the gluten-free bread (Table 1 and Table 2), probably due to a reduction in the crust cracks, the dry appearance, and the crumbly texture, characteristics of gluten-free bread [27].

For light crust color, 25% AK flour bread was more frequent. The highest frequency was found in 12.5% AK flour bread for light crumb color, and dark crumb color was more observed in 50% bread. In our previous instrumental evaluation [16], the 50% AK flour bread differed the most from the control bread, obtaining one of the lowest averages for chroma, indicating a loss of crumb color purity. The sample considered the most beautiful and bright on CATA was the 12.5% bread.

Figure 2 and Figure 3 show the correlation of the evaluated attributes with the evaluators’ preferences. A graph is generated that presents the impact of the assessed attributes (CATA) on the sensorial acceptance of the consumers. A descriptive symmetric map was generated from CATA. The map generated by the correspondence analysis elucidates 91.98% of the variation in the two dimensions analyzing all participants (*n* = 110) (Figure 2). Considering the gluten-free bread consumers, the results were similar (88.96% of the variation in the two dimensions). According to the map, 12.5% of AK bread is in the lower-left quadrant, close to light crust color, light crumb color, soft and moist texture, cohesive, pleasant, and shiny characteristics. Control bread is located in the upper right quadrant next to characteristics such as dry appearance, dry and grainy texture, dark crust, and salty. The remaining 25%, 37.5%, and 50% bread samples are located in the upper left quadrant, where characteristics such as fish aroma, yeast aroma, dark crumb, blackberry, yeast flavor, and unpleasant flavor can be observed. The proximity of these bread samples on the map demonstrates similarities between them.

A previous study [16] on AK flour gluten-free bread’s nutritional and physicochemical properties showed that the best formulations were prepared with up to 37.5% AK flour concentrations. Additionally, the authors showed that the 12.5% AK flour presented the highest protein content among AK flour gluten-free bread samples, which could affect technological and sensory characteristics in the absence of gluten. Our study showed that the 12.5% AK flour gluten-free bread sample presented the best sensory profile among AK gluten-free bread samples. It is confirmed to be a good alternative to replace gluten-free flour in gluten-free bread in more significant amounts than previously studied as an additive.

This research presents, as a limitation, the absence of the descriptive analysis (DA), a more robust and reliable evaluation tool when precise definitions and quantification of the sensory attributes of products are required [29,30]. However, CATA was applied, since it is a rapid sensory profiling tool that can be applied by non-trained panelists, spending less time and money than descriptive analysis [29,30]. Using descriptive analysis, further research may be applied to evaluate AK gluten-free bread with a trained sensory panel.

## 5. Conclusions

*Amorphophallus konjac* flour interfered with the sensory characteristics of gluten-free bread. Adding up to 12.5% of AK flour (in partial replacement of gluten-free flour) was feasible on the gluten-free bread formulation used in this study. The most desirable attributes in the studied bread were aroma, pleasant taste, beautiful appearance, and clear crumb, found in gluten-free bread with 12.5% AK flour. In this way, the study demonstrates that it is feasible to use AK flour in higher concentrations than being used as an additive, up to 12.5%, helping to improve sensory characteristics of gluten-free products. Further studies should be conducted to evaluate the glycemic index of these gluten-free bread, determine the shelf life, and determine the purchase intention of the products so that they can go to the consumer market.

## Figures and Tables

**Table 1:** Mean and standard deviation of gluten-free bread samples’ acceptance (9-point hedonic scale; *n* = 110 tasters).

*Amorphophallus konjac* (AK) Flour (% of Flour Replacement)	Appearance	Flavor	Aroma	Texture	Global Acceptance
0	7.28 ± 1.81 ^b^	7.15 ± 1.74 ^a^	7.07 ± 1.65 ^a^	5.60 ± 2.14 ^a^	6.78 ± 1.77 ^b^
12.5	8.09 ± 1.85 ^a^	7.36 ± 1.55 ^a^	6.95 ± 1.80 ^a^	7.39 ± 1.45 ^b^	7.53 ± 1.22 ^a^
25	6.67 ± 1.72 ^bc^	5.40 ± 2.03 ^b^	5.70 ± 1.88 ^b^	5.47 ± 2.04 ^a^	5.66 ± 1.80 ^c^
37.5	7.19 ± 1.64 ^b^	5.57 ± 2.14 ^b^	6.13 ± 1.97 ^b^	5.64 ± 2.04 ^a^	5.82 ± 1.99 ^c^
50	6.46 ± 1.89 ^c^	4.65 ± 2.07 ^c^	5.64 ± 1.99 ^b^	4.92 ± 2.20 ^a^	5.07 ± 1.99 ^c^

In the columns, means with the same letters do not differ statistically by Tukey’s test (*p* < 0.05).

**Table 2:** Percentage of acceptance of gluten-free bread formulations among consumers (*n* = 43) and non-consumers of gluten-free bread (*n* = 67).

Gluten-Free Bread Consumers (% of Acceptance)					
*Amorphophallus konjac* (AK) Flour (% of Flour Replacement)	Appearance %	Flavor %	Aroma %	Texture %	Global Acceptance %
0	81.39	95.34	86.05	60.46	83.72
12.5	95.35	90.70	76.75	88.37	93.03
25	72.09	48.84	55.81	58.14	67.44
37.5	93.02	55.81	65.12	55.81	67.44
50	76.75	41.86	48.84	37.21	48.84
Gluten-free bread non-consumers (% of acceptance)					
0	85.07	74.63	73.13	50.74	73.13
12.5	97.02	82.58	76.11	86.57	95.52
25	76.12	47.76	44.77	50.75	49.25
37.5	80.60	50.74	61.19	55.22	56.71
50	61.19	29.85	49.25	37.31	38.81

**Table 3:** Absolute frequencies of sensory attributes checked for the different *Amorphophallus konjac* (AK) gluten-free bread among all consumers (Group I; *n* = 110).

	Attributes	Control	12.5	25	37.5	50	*p*-Values (Cochran’s Q)
Appearance	Dark Crust Color	18 ^ab^	42 ^c^	8 ^a^	13 ^a^	31 ^bc^	<0.0001
	Dry Crust Appearence	5 ^a^	60 ^c^	20 ^b^	9 ^ab^	16 ^ab^	<0.0001
	Light Crust Color	56 ^bc^	33 ^a^	77 ^d^	74 ^cd^	42 ^ab^	<0.0001
	Light Crumb Color	78 ^b^	59 ^a^	68 ^ab^	72 ^ab^	58 ^a^	0.003
	Dark Crumb Color	9 ^a^	7 ^a^	15 ^ab^	10 ^a^	30 ^b^	<0.0001
	Pretty	66 ^c^	50 ^bc^	28 ^a^	33 ^ab^	25 ^a^	<0.0001
	Shiny	10 ^a^	6 ^a^	6 ^a^	3 ^a^	1 ^a^	0.031
Aroma	Egg Scent	6 ^a^	7 ^a^	14 ^a^	11 ^a^	13 ^a^	0.143
	Fried Food Scent	6 ^a^	13 ^a^	12 ^a^	9 ^a^	10 ^a^	0.376
	Seafood Scent	13 ^b^	1 ^a^	16 ^b^	18 ^b^	26 ^b^	<0.0001
	Yeast Scent	28 ^a^	30 ^a^	33 ^a^	29 ^a^	34 ^a^	0.152
	Pleasant Scent	65 ^bc^	77 ^c^	43 ^a^	49 ^ab^	35 ^a^	<0.0001
Flavor	Seafood Flavor	8 ^ab^	1 ^a^	14 ^b^	17 ^b^	21 ^b^	<0.0001
	Yeast Flavor	19 ^ab^	13 ^a^	34 ^b^	26 ^ab^	27 ^ab^	0.003
	Unpleasant Flavor	3 ^a^	6 ^a^	27 ^b^	34 ^b^	39 ^b^	<0.0001
	Pleasant Flavor	80 ^b^	73 ^b^	37 ^a^	36 ^a^	28 ^a^	<0.0001
	Bitter	2 ^a^	3 ^a^	4 ^a^	3 ^a^	5 ^a^	0.804
	Sour	2 ^a^	2 ^a^	1 ^a^	2 ^a^	1 ^a^	0.50
	Sweet Flavor	23 ^bc^	36 ^c^	4 ^a^	10 ^ab^	6 ^a^	<0.0001
	Astringent	1 ^a^	1 ^a^	7 ^a^	9 ^a^	4 ^a^	0.011
	Savory Flavor	18 ^a^	34 ^b^	19 ^ab^	12 ^a^	11 ^a^	<0.0001
Texture	Moist	34 ^b^	1 ^a^	25 ^b^	34 ^b^	31 ^b^	<0.0001
	Stiff	2 ^a^	50 ^c^	15 ^b^	8 ^ab^	5 ^ab^	<0.0001
	Sticky	6 ^a^	1 ^a^	26 ^b^	28 ^b^	49 ^c^	<0.0001
	Cohese	15 ^a^	5 ^a^	7 ^a^	8 ^a^	5 ^a^	0.010
	Rubbery	10 ^a^	1 ^a^	57 ^b^	47 ^b^	53 ^b^	<0.0001
	Soft	82 ^c^	3 ^a^	40 ^b^	39 ^b^	34 ^b^	<0.0001
	Compact	21 ^a^	26 ^a^	22 ^a^	14 ^a^	15 ^a^	0.155
	Buttery	25 ^b^	26 ^b^	6 ^a^	16 ^ab^	8 ^a^	<0.0001
	Grainy	2 ^a^	40 ^d^	9 ^ab^	15 ^bc^	24 ^cd^	<0.0001
	Dry	8 ^a^	76 ^b^	9 ^a^	3 ^a^	6 ^a^	<0.0001

Frequencies with which sensory terms were checked in the CATA question. Different letters in the same row indicate significant differences (Mcnemar Bonferroni Multiple pairwise comparison; *p* < 0.05).

**Table 4:** Absolute frequencies of sensory attributes checked for the different AK gluten-free bread among frequent consumers of gluten-free bread (Group II; *n* = 43).

	Attributes	Control	12.5	25	37.5	50	*p*-Values Cochran’s Q
Appearence	Dark Crust Color	7 ^a^	17 ^a^	4 ^a^	6 ^a^	14 ^a^	0.001
	Dry Crust Appearence	1 ^a^	29 ^b^	8 ^a^	2 ^a^	6 ^a^	<0.0001
	Light Crust Color	23 ^ab^	11 ^a^	29 ^b^	29 ^b^	12 ^a^	<0.0001
	Light Crumb Color	29 ^a^	28 ^a^	24 ^a^	27 ^a^	19 ^b^	0.066
	Dark Crumb Color	4 ^ab^	2 ^a^	7 ^ab^	4 ^ab^	15 ^b^	<0.0001
	Pretty	27 ^b^	21 ^a^	11 ^a^	13 ^a^	10 ^a^	<0.0001
	Shiny	2 ^a^	1 ^a^	2 ^a^	0 ^a^	0 ^a^	0.406
Aroma	Egg Scent	2 ^a^	2 ^a^	3 ^a^	0 ^a^	3 ^a^	0.532
	Fried Food Scent	3 ^a^	3 ^a^	6 ^a^	5 ^a^	4 ^a^	0.519
	Seafood Scent	6 ^ab^	0 ^a^	9 ^ab^	9 ^ab^	13 ^b^	0.001
	Yeast Scent	11 ^a^	8 ^a^	12 ^a^	13 ^a^	13 ^a^	0.639
	Pleasant Scent	25 ^b^	32 ^c^	17 ^a^	18 ^a^	13 ^d^	<0.0001
Flavor	Seafood Flavor	5 ^ab^	0 ^a^	11 ^b^	11 ^b^	11 ^b^	0.001
	Yeast Flavor	7 ^a^	2 ^a^	10 ^a^	10 ^a^	12 ^a^	0.038
	Unpleasant Flavor	1 ^a^	1 ^a^	8 ^ab^	13 ^b^	12 ^b^	<0.0001
	Pleasant Flavor	31 ^b^	32 ^b^	13 ^a^	13 ^a^	11 ^a^	<0.0001
	Bitter	1 ^a^	1 ^a^	3 ^a^	0 ^a^	2 ^a^	0.406
	Sour	0 ^a^	1 ^a^	1 ^a^	1 ^a^	0 ^a^	0.736
	Sweet Flavor	10 ^ab^	12 ^b^	1 ^a^	3 ^ab^	1 ^ab^	<0.0001
	Astringent	0 ^a^	0 ^a^	3 ^a^	4 ^a^	3 ^a^	0.083
	Savory Flavor	6 ^ab^	13 ^b^	7 ^ab^	4 ^ab^	3 ^a^	0.003
Texture	Moist	11 ^b^	0 ^a^	10 ^b^	13 ^b^	13 ^b^	0.001
	Stiff	2 ^a^	20 ^b^	6 ^ab^	4 ^a^	2 ^a^	<0.0001
	Sticky	3 ^ab^	0 ^a^	9 ^ab^	10 ^b^	15 ^b^	<0.0001
	Cohese	5 ^a^	1 ^a^	1 ^a^	1 ^a^	2 ^a^	0.056
	Rubbery	5 ^a^	1 ^a^	22 ^b^	20 ^b^	21 ^b^	<0.0001
	Soft	32 ^c^	2 ^a^	16 ^b^	12 ^ab^	12 ^ab^	<0.0001
	Compact	8 ^a^	9 ^a^	8 ^a^	6 ^a^	4 ^a^	0.573
	Buttery	7 ^a^	11 ^a^	3 ^a^	6 ^a^	1 ^a^	0.013
	Grainy	1 ^a^	14 ^b^	3 ^ab^	6 ^b^	11 ^b^	0.000
	Dry	2 ^a^	30 ^b^	3 ^a^	2 ^a^	2 ^a^	<0.0001

Frequencies with which sensory terms were checked in the CATA question. Different letters in the same row indicate a significant difference (Mcnemar Bonferroni Multiple pairwise comparisons; *p* < 0.05).

## Data Availability

Data is contained within the article or supplementary material.

## Supplementary Materials

The following supporting information can be downloaded at: https://www.mdpi.com/article/10.3390/foods11101379/s1, Table S1. Formulations of gluten-free bread with and without the addition of konjac flour.
